# When Mood and Memory Move Together: Psychodynamic/Relational Factors in Late-Life Pseudodementia

**DOI:** 10.1192/j.eurpsy.2026.11585

**Published:** 2026-07-31

**Authors:** S. Castelao-Almodovar, A. P. Balaguer, E. G. Benito

**Affiliations:** Centro de Salud Mental El Escorial, El Escorial, Spain

## Abstract

**Introduction:**

Pseudodementia denotes potentially reversible cognitive impairment secondary to depression and may overlap with neurodegenerative presentations in late life. Beyond mood, psychodynamic, relational, and broader emotional processes can shape the salience and reporting of cognitive symptoms, treatment engagement, and functional impact.

**Objectives:**

To describe a longitudinal single-case of late-life depression with a pseudodementia presentation, examining temporal co-variation between mood and mnestic complaints, and exploring how psychodynamic/relational/emotional processes observed during routine follow-up may relate to symptom expression and treatment engagement.

**Methods:**

Observational case report in community psychiatry (April 2024–September 2025). Baseline neuropsychology was consistent with amnestic Mild Cognitive Impairment (MoCA 28/30; impaired verbal learning/recall; preserved visual memory; executive inefficiencies; activities of daily living intact). Pharmacological management included a switch from escitalopram to vortioxetine with dose optimization. Psychiatric follow-up adopted a psychodynamic stance that explicitly considered emotional meaning and the patient’s relational context, with couple participation as clinically indicated. Cognitive rehabilitation was recommended. Outcomes were tracked through serial clinical interviews and session process notes.

**Results:**

Optimization of vortioxetine was associated with progressive mood improvement and a parallel reduction in memory failures. Fluctuations in cognitive complaints tended to cluster around emotionally salient or relationally tense contexts; this pattern emerged as a clinical impression and warrants systematic study. Work that addressed emotional meanings, acceptance of limitations, and patterns of relating coincided with better tolerance of errors and greater use of compensatory strategies. In contrast, adherence to cognitive rehabilitation was poor because the patient did not perceive benefit, whereas motivation and engagement were higher for psychodynamic/relational work. Transient autonomic symptoms occurred during emotionally intensive sessions (brief dizziness with normal blood pressure). Despite clinical gains, the neuropsychological profile remained compatible with amnestic MCI; daily functioning was preserved. Symptoms have not remitted, and the patient remains in active treatment.

**Conclusions:**

In late-life depression with a pseudodementia phenotype, antidepressant treatment improved mood, but integrating a psychodynamic-informed clinical stance and attention to the relational/emotional context was associated with additional benefits in subjective cognition and behavior. The observed links between affective–relational processes and cognitive complaints are hypothesis-generating and should be evaluated in controlled studies to delineate mechanisms and identify who benefits most from this integrative approach.

**Disclosure of Interest:**

None Declared

